# Glucocorticoid dose at 26 weeks and subsequent relapse in anti-ARS antibody-positive antisynthetase syndrome under concomitant immunosuppressive therapy: a retrospective cohort study

**DOI:** 10.3389/fmed.2026.1983345

**Published:** 2026-10-29

**Authors:** Ayaka Inoue, Takashi Yamane, Atsushi Onoe, Noriaki Yasuda, Takahisa Ohnishi

**Affiliations:** Department of Rheumatology, Kakogawa Central City Hospital, Kakogawa, Hyogo, Japan

**Keywords:** antisynthetase syndrome, glucocorticoid tapering, immunosuppressive therapy, interstitial lung disease, prednisolone, relapse

## Abstract

**Background:**

Glucocorticoids (GCs) remain central to antisynthetase syndrome (ASSD) treatment, but relapse during tapering and GC-related adverse effects remain important concerns. Because GC-related morbidity may emerge early, minimizing GC exposure while maintaining disease control is important. Whether the GC dose during early tapering is associated with subsequent relapse remains unclear. We investigated whether a GC dose of ≤5 mg/day at approximately 6 months was associated with subsequent relapse in anti-aminoacyl-tRNA synthetase (ARS) antibody-positive ASSD receiving concomitant immunosuppressive therapy.

**Methods:**

We retrospectively analyzed 45 patients with anti-ARS antibody-positive ASSD treated with systemic GCs and concomitant immunosuppressive therapy. We performed a week-26 landmark analysis, representing approximately 6 months after treatment initiation, among patients who remained relapse-free and under observation through the landmark. Subsequent relapse-free survival was compared between patients receiving GC ≤5 and >5 mg/day in prednisolone equivalents at the landmark.

**Results:**

During overall follow-up, 26 of 45 patients (58%) experienced relapse, whereas only 4 (8.9%) relapsed before week 26. Among 41 patients who remained relapse-free and under observation through the landmark, 17 were receiving GC ≤5 mg/day and 24 were receiving >5 mg/day. During post-landmark follow-up, 22 patients experienced subsequent relapse. Patients receiving GC ≤5 mg/day at week 26 had higher subsequent relapse-free survival than those receiving >5 mg/day (log-rank *p* = 0.007). In multivariable analyses, GC ≤5 mg/day at week 26 was associated with a lower risk of subsequent relapse after adjustment for Raynaud phenomenon and initial GC dose (HR 0.27, 95% CI 0.08–0.86; *p* = 0.027) and after adjustment for Raynaud phenomenon and anti-Jo-1 positivity (HR 0.32, 95% CI 0.10–0.99; *p* = 0.047).

**Conclusion:**

Among patients with anti-ARS antibody-positive ASSD receiving concomitant immunosuppressive therapy who remained relapse-free and under observation through the week-26 landmark, a GC dose ≤5 mg/day was associated with a lower risk of subsequent relapse. Although these findings should not be generalized to all patients or interpreted as establishing an optimal tapering target, the GC dose at this landmark may serve as a clinically useful marker of early disease control or treatment response. Prospective studies are warranted to validate its clinical significance.

## Introduction

Idiopathic inflammatory myopathies (IIMs) are a heterogeneous group of autoimmune diseases with distinct clinical phenotypes and prognoses that are strongly influenced by autoantibody profiles. Anti-synthetase syndrome (ASSD) is a major clinical subset characterized by anti-aminoacyl-tRNA synthetase (ARS) antibodies and manifestations including myositis, interstitial lung disease (ILD), inflammatory arthritis, Raynaud phenomenon, mechanic's hands, and fever (1–4).

Although ASSD generally responds to glucocorticoid (GC) therapy, relapse during dose tapering remains a major clinical challenge (5, 6). Because relapse is common with GC monotherapy, immunosuppressive agents are frequently used in combination with GCs, particularly in patients with ILD (7, 8). Prolonged GC exposure is associated with substantial adverse effects, and minimizing cumulative GC exposure while maintaining disease control is therefore an important therapeutic goal (9–11). Even low-dose GC therapy may contribute to clinically relevant adverse events when continued for prolonged periods (12–14). Importantly, GC-related morbidity may emerge early during treatment, and bone loss has been reported to develop within the first 6 months after diagnosis in patients with IIM (15, 16). A GC dose equivalent to 5 mg/day of prednisolone represents a low-dose level of particular clinical interest because adverse effects may persist even at low doses. Concomitant immunosuppressive therapy may facilitate GC tapering and improve disease control in ASSD. However, relapse remains common during long-term follow-up (6, 7, 17). Thus, an important clinical challenge is to minimize GC exposure while identifying patients who can maintain long-term disease control. However, it remains unclear whether achievement of a low GC dose during the early treatment course is associated with subsequent relapse in patients with ASSD receiving concomitant immunosuppressive therapy.

In this study, we retrospectively evaluated whether a GC dose of ≤5 mg/day at week 26 was associated with subsequent relapse among anti-ARS antibody-positive patients with ASSD receiving concomitant immunosuppressive therapy who remained relapse-free and under observation through week 26.

## Materials and methods

### Patients and study design

Between July 2016 and September 2024, we identified patients with positive anti-ARS antibodies at our institution. The administrative chart review was conducted during September 2024. For patients without earlier relapse or censoring, follow-up was censored at the latest documented clinical visit available at the time of the chart review. Anti-ARS antibodies were measured using a commercially available enzyme immunoassay (EIA; LSI Medience Corporation, Tokyo, Japan). Anti-Jo-1 antibodies were measured separately using a chemiluminescent enzyme immunoassay (CLEIA; LSI Medience Corporation), with a positivity threshold of ≥10 U/mL. Patients with a positive anti-ARS antibody result and a negative anti-Jo-1 antibody result were classified as the non-Jo-1 group. Individual non-Jo-1 anti-ARS specificities, including PL-7, PL-12, EJ, OJ, and KS, were assessed only in a limited number of patients when clinically indicated and were therefore not systematically available for the entire cohort. Patients diagnosed with ASSD who received systemic GC therapy and had at least 26 weeks of follow-up were eligible for inclusion. Patients were excluded if they were untreated or if GC dosing and immunosuppressive treatment were primarily determined by a coexisting connective tissue disease. The latter was defined as the use or intensification of immunosuppressive and/or biologic therapy for clinical manifestations attributable to the coexisting disease rather than to ASSD. The initial weight-adjusted GC dose was calculated for each patient by dividing the initial prednisolone-equivalent GC dose (mg/day) by body weight (kg). Patients were excluded if treatment was primarily directed at another connective tissue disease, if remission-induction treatment for ASSD had been initiated before the study inclusion period, if they were managed without systemic treatment, or if sufficient information for assessment through week 26 was unavailable. Treatment was considered primarily driven by another connective tissue disease when the coexisting connective tissue disease, rather than ASSD manifestations, was judged by the treating physician to be the principal indication for systemic immunosuppressive treatment. For the week-26 landmark analysis, patients who experienced relapse before week 26 were excluded. Thus, the landmark analysis was restricted to patients who remained relapse-free and under observation through week 26, with follow-up for subsequent relapse beginning at the landmark.

ASSD was defined according to the Connors criteria as anti-ARS antibody positivity together with at least one characteristic clinical manifestation (18). All GC doses were expressed as prednisolone-equivalent doses.

This study was conducted in accordance with the Declaration of Helsinki and was approved by the Ethics Committee of Kakogawa Central City Hospital (Approval No. 2024-49). The requirement for informed consent was waived by the Ethics Committee because of the retrospective nature of the study and the use of anonymized clinical data.

### Treatment and definition of relapse

GC tapering was performed at the discretion of the treating physician after confirmation of clinical stability. In patients treated with tacrolimus, the dose was generally adjusted to target a whole-blood trough concentration of 5–10 ng/mL, with further adjustment according to disease activity and tolerability, as previously described (19). Tacrolimus (TAC) dosing was adjusted in routine clinical practice with reference to trough concentrations. Because the timing of trough measurements was not standardized and measurements around the week-26 landmark were not uniformly available, actual TAC trough concentrations were not included as a quantitative landmark exposure variable. The timing of immunosuppressive therapy was assessed as the interval from initiation of GC treatment to initiation of immunosuppressive therapy. Immunosuppressive regimens and treatment changes before week 26, as well as treatment status at the week-26 landmark, were retrospectively obtained from the medical records.

Relapse was defined as clinically attributable worsening of ASSD manifestations requiring an increase in the GC dose and/or initiation or intensification of immunosuppressive therapy. Because prespecified standardized organ-specific criteria were not applied in this retrospective study, relapse was determined by the treating physician based on clinical manifestations together with relevant laboratory and/or imaging findings. ILD relapse was assessed by worsening interstitial abnormalities on chest CT accompanied by worsening respiratory symptoms, with alternative causes, particularly infection, excluded based on the clinical context, microbiological testing when indicated, and response to antimicrobial therapy in uncertain cases. Myositis relapse was assessed primarily by increased serum CK levels and/or worsening muscle symptoms, with alternative causes considered from the clinical history and course. Arthritis relapse was assessed by recurrent or worsening joint swelling and pain, supported by inflammatory markers, and skin disease relapse by new or worsening cutaneous manifestations characteristic of dermatomyositis that persisted without spontaneous improvement. Formal remission was not required, but persistent activity without preceding improvement or stability was not classified as relapse; relapse required subsequent worsening after a period of clinical improvement or stability.

### Week-26 landmark analysis

To evaluate the association between GC dose during the early treatment course and subsequent relapse, survival analyses were performed using week 26 after treatment initiation as the landmark. Week 26 represents approximately 6 months after treatment initiation and was selected as a clinically relevant but partly pragmatic time point for assessing GC dose during early tapering. Patients who relapsed before week 26 were excluded, and the landmark analysis was restricted to those who remained relapse-free and under observation through the landmark.

Time zero was defined as week 26, and the outcome was the first relapse occurring thereafter. Patients were followed until first relapse or censoring at the last confirmed follow-up, transfer to another institution, or death without prior relapse, whichever occurred first. Kaplan–Meier and Cox proportional hazards analyses used the same landmark population, event definition, time origin, and censoring rules. *P* values for Cox proportional hazards analyses were based on the Wald test.

At the landmark, patients were classified according to their GC dose (≤5 vs. >5 mg/day in prednisolone equivalents). ILD activity was assessed using clinical symptoms, chest CT findings, and KL-6 levels, whereas myositis activity was assessed using clinical findings and serial CK levels. ILD extent was not systematically quantified using a standardized radiological scoring system, and pulmonary function tests were not performed at standardized time points in a sufficient proportion of patients to permit meaningful group-level comparison.

Relapse-free survival after week 26 was estimated using the Kaplan–Meier method with 95% confidence intervals and compared between the GC-dose groups using the log-rank test.

### Statistical analysis

In the week-26 landmark cohort, demographic and clinical characteristics, anti-ARS antibody status, initial treatment, available laboratory and treatment characteristics at week 26, and post-landmark follow-up duration were compared between patients receiving GC ≤5 and >5 mg/day. Continuous variables were compared using the Mann–Whitney U test, and categorical variables were compared using Fisher's exact test.

Missing data were not imputed, and available-case denominators were used for variables with missing data. Cox proportional hazards analyses were performed in the week-26 landmark cohort (*n* = 41), with time from week 26 to first subsequent relapse as the time scale. There were no missing data for the covariates included in the multivariable Cox models. Therefore, both models included all 41 patients.

Given the limited number of post-landmark relapse events, the number of covariates in each multivariable model was restricted. Because Raynaud phenomenon was uncommon, sensitivity analyses excluding this variable were performed, with GC ≤5 mg/day at week 26 separately adjusted for initial GC dose and anti-Jo-1 positivity. The week-26 GC dose was also analyzed as a continuous variable in a univariable Cox model. The proportional hazards assumption was assessed by testing interactions between each covariate and the logarithm of time, with no significant violations detected. Continuous variables are presented as mean ± standard deviation or median [interquartile range (IQR)], as appropriate, and categorical variables as number (%). Statistical analyses were performed using SPSS Statistics version 26 (IBM Corp., Armonk, NY, USA). All tests were two-sided, and *p* < 0.05 was considered statistically significant.

## Results

### Patient characteristics and treatment in the baseline cohort

Among 96 patients screened, 51 were excluded: 11 because treatment was primarily driven by another connective tissue disease, 15 because remission-induction treatment had already been initiated before the study inclusion period, 12 because they were managed without systemic treatment, and 13 because sufficient information for assessment through week 26 was unavailable (early death, *n* = 6; loss to follow-up, *n* = 2; unavailable treatment data, *n* = 5). The remaining 45 patients constituted the overall study cohort. Four patients experienced relapse before week 26 and were excluded from the landmark analysis, leaving 41 patients who remained relapse-free and under observation through week 26 for the primary landmark analysis (Figure 1).

**Figure 1:**
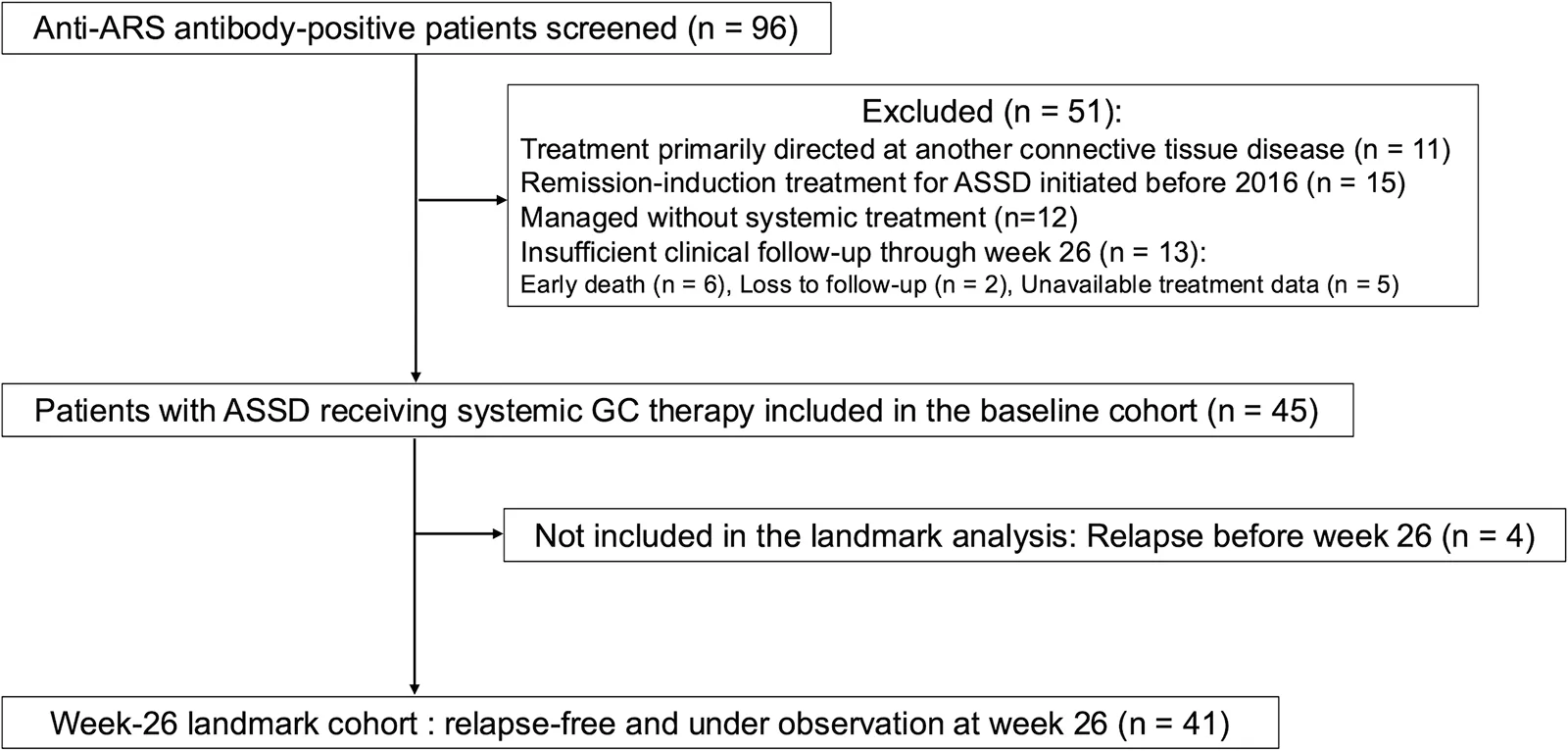
Flow diagram of patient selection and the week-26 landmark analysis. Of 96 anti-ARS antibody-positive patients screened, 51 were excluded and 45 were included in the baseline cohort. Four patients who experienced relapse before week 26 were not included in the landmark analysis, leaving 41 patients in the week-26 landmark cohort.

Baseline characteristics and initial treatment of the 45 patients are summarized in Table 1. During the overall follow-up period, 26 of the 45 patients (58%) experienced relapse. The mean age was 65 ± 13 years, 25 patients (56%) were women, and the median follow-up duration was 48 months (IQR, 26–81). ILD was present in 40 patients (89%). Eleven patients (24%) were positive for anti-Jo-1 antibodies, and 10 (22%) were positive for anti-SS-A (Ro60) antibodies. Clinical manifestations included myositis in 20 patients (44%), arthritis in 20 (44%), mechanic's hands in 29 (64%), and Raynaud phenomenon in 6 (13%). All six patients with Raynaud phenomenon experienced relapse during the overall follow-up period. Initial treatment included methylprednisolone pulse therapy in 18 patients (40%). The mean initial GC dose was 42 ± 16 mg/day in prednisolone equivalents (0.74 ± 0.23 mg/kg/day). All patients received concomitant immunosuppressive therapy, with tacrolimus used in 43 patients (96%). All patients receiving combination immunosuppressive therapy before week 26 were treated with tacrolimus plus intravenous cyclophosphamide. The immunosuppressive regimen was changed before week 26 in one patient, from cyclosporine A to tacrolimus because of liver injury.

**Table 1:** Clinical characteristics and initial treatment of patients with anti-ARS antibody-positive ASSD in the baseline cohort (*n* = 45).

Variable	Overall (*n* = 45)
Demographic and follow-up characteristics	
Age, years	65 ± 13
Female	25 (56)
Follow-up duration, months	48 [26–81]
Anti-ARS antibody status	
Anti-Jo-1	11 (24)
Non-Jo-1	34 (76)
Additional autoantibodies	
Anti-SS-A (Ro60) positive	10 (22)
Clinical features	
Interstitial lung disease	40 (89)
Myositis	20 (44)
Arthritis	20 (44)
Mechanic's hands	29 (64)
Raynaud phenomenon	6 (13)
Gottron's sign	19 (42)
Fever	13 (29)
Laboratory findings	
CRP, mg/dL	1.00 [0.21–3.56]
CK, U/L	138 [70–825]
KL-6, U/mL	837 [451–1652]
Initial remission-induction treatment	
Methylprednisolone pulse therapy	18 (40)
Initial GC dose, mg/day	42 ± 16
Concomitant IS therapy	45 (100)
TAC monotherapy	36 (80)
CyA monotherapy	1 (2)
MTX monotherapy	1 (2)
Combination IS therapy	7 (16)

Data are presented as mean ± SD, median [IQR], or number (%). Non-Jo-1 was defined as anti-ARS antibody positivity with a negative anti-Jo-1 antibody result; individual non-Jo-1 anti-ARS specificities were not systematically determined. Initial treatment refers to remission-induction therapy administered at diagnosis. Combination IS therapy includes tacrolimus plus mycophenolate mofetil, methotrexate, cyclophosphamide, or rituximab. GC doses are expressed as prednisolone equivalents. ASSD, anti-synthetase syndrome; ARS, aminoacyl-tRNA synthetase; CRP, C-reactive protein; CK, creatine kinase; KL-6, Krebs von den Lungen-6; GC, glucocorticoid; IS, immunosuppressant; TAC, tacrolimus; CyA, cyclosporine A; MTX, methotrexate; MMF, mycophenolate mofetil; RTX, rituximab; CY, cyclophosphamide.

### Week-26 landmark analysis

Among the 41 patients in the week-26 landmark cohort, 22 experienced a subsequent first relapse: 4 of 17 patients (24%) in the GC ≤5 mg/day group and 18 of 24 patients (75%) in the GC >5 mg/day group. The median post-landmark follow-up duration was 78 weeks (IQR, 48–164) and 72 weeks (IQR, 22–153), respectively (*p* = 0.278). Among patients without relapse, 13 in the GC ≤5 mg/day group and 6 in the GC >5 mg/day group were censored. In the GC ≤5 mg/day group, censoring occurred at administrative follow-up in 10 patients, death without prior relapse in one patient, and transfer without prior relapse in two patients. In the GC >5 mg/day group, five patients were administratively censored and one died without prior relapse.

Kaplan–Meier analysis showed higher subsequent relapse-free survival in the GC ≤5 mg/day group than in the GC >5 mg/day group (log-rank *p* = 0.007; Figure 2).

**Figure 2:**
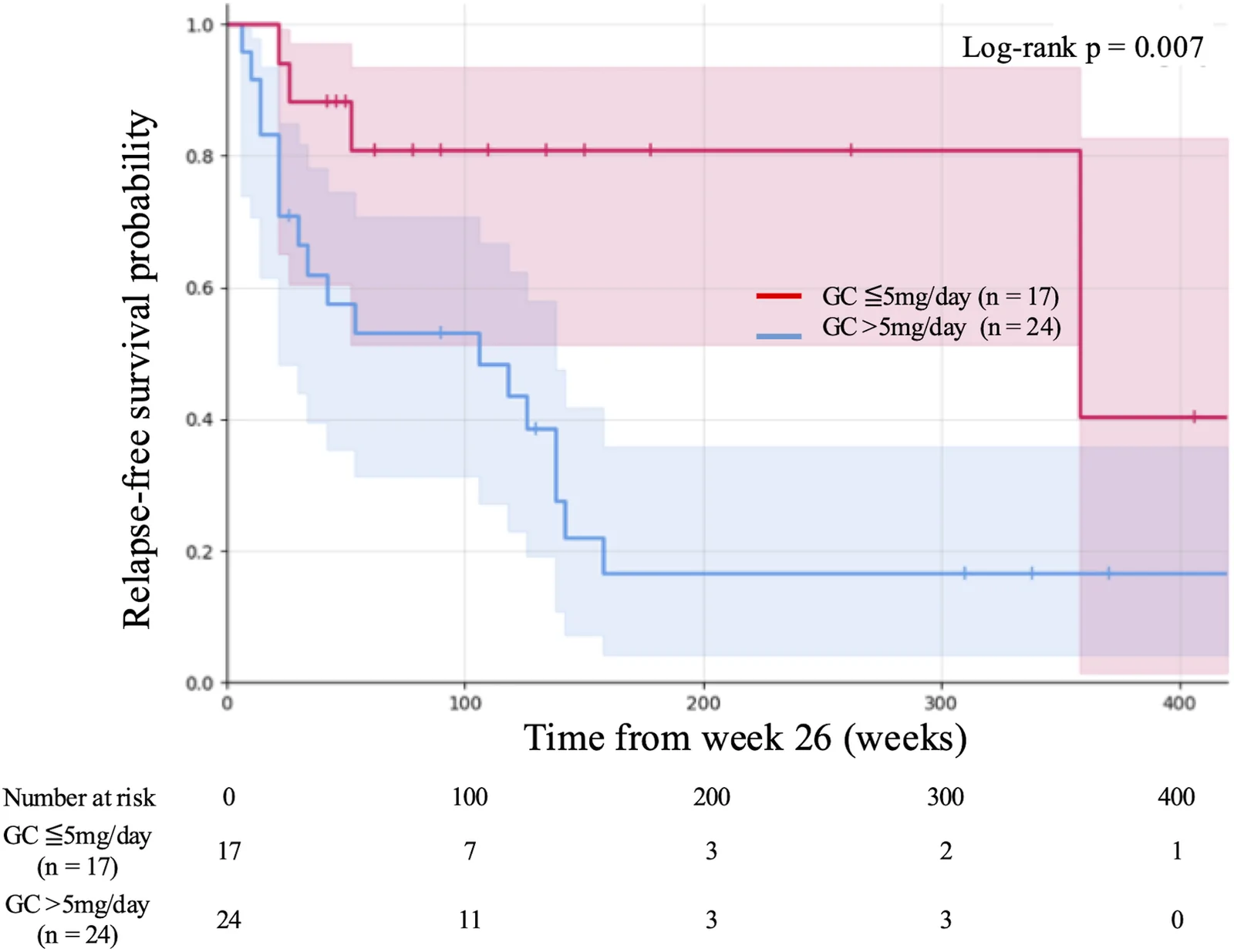
Relapse-free survival according to glucocorticoid dose at week 26. Kaplan–Meier curves showing relapse-free survival after the week-26 landmark according to GC dose at week 26 (≤5 vs. >5 mg/day in prednisolone equivalents). The analysis included 41 patients who remained relapse-free and under observation through week 26. Relapse-free survival was significantly higher in patients with GC ≤5 mg/day than in those with GC >5 mg/day (log-rank *p* = 0.007). Shaded areas indicate 95% confidence intervals. Tick marks indicate censored observations. Numbers below the graph indicate patients at risk.

### Week-26 group comparison

Characteristics of the 41 patients stratified by GC dose at week 26 are shown in Table 2. Patients in the GC ≤5 mg/day group were older than those in the >5 mg/day group (69 ± 13 vs. 63 ± 12 years; *p* = 0.044), whereas mechanic's hands were less frequent (47% vs. 79%; *p* = 0.048). Initial GC dose was numerically lower in the GC ≤5 mg/day group (37 ± 16 vs. 45 ± 16 mg/day; *p* = 0.055), and combination immunosuppressive therapy was observed only in the >5 mg/day group. Immunosuppressive therapy was initiated a median of 7 days (IQR, 0–20) after initiation of GC treatment. The median maximum TAC dose through week 26 was 3 mg/day (IQR, 2–3) in both groups. At week 26, CRP, CK, and KL-6 levels were not significantly different between the groups. Raynaud phenomenon was present in five patients, all of whom were in the GC >5 mg/day group and subsequently experienced relapse. The median post-landmark follow-up duration was 78 weeks (IQR, 48–164) in the GC ≤5 mg/day group and 72 weeks (IQR, 22–153) in the >5 mg/day group (*p* = 0.278). Subsequent relapse occurred in 4 of 17 patients (24%) and 18 of 24 patients (75%), respectively.

**Table 2:** Clinical characteristics of patients in the week-26 landmark cohort according to glucocorticoid dose.

Variable	GC ≤5 mg/day at week 26 (*n* = 17)	GC >5 mg/day at week 26 (*n* = 24)	*p* value
**Demographic characteristics**			
Age, years	69 ± 13	63 ± 12	0.044
Female	7 (41)	16 (67)	0.125
**Autoantibody status**			
Anti-Jo-1 positive	4 (24)	7 (29)	0.736
**Clinical features**			
Interstitial lung disease	13 (76)	23 (96)	0.141
Myositis	7 (41)	11 (46)	1.00
Arthritis	7 (41)	12 (50)	0.752
Mechanic's hands	8 (47)	19 (79)	0.048
Raynaud phenomenon	0 (0)	5 (21)	0.065
**Initial remission-induction treatment**			
Methylprednisolone pulse therapy	6 (35)	10 (42)	0.753
Initial GC dose, mg/day	37 ± 16	45 ± 16	0.055
TAC monotherapy	15 (88)	18 (75)	0.433
Maximum TAC dose, mg/day*	3 (2–3)	3 (2–3)	0.312
Combination IS therapy	0 (0)	5 (21)	0.065
**Characteristics at week 26**			
CRP, mg/dL	0.12 [0.03–0.22]	0.15 [0.02–0.20]	0.314
CK, U/L	51 [34–76]	60 [45–93]	0.464
KL-6, U/mL	338 [274–718]	481 [303–685]	0.725
TAC monotherapy	16 (94)	18 (75)	0.207
Combination IS therapy	0 (0)	5 (21)	0.065
**Follow-up after the landmark**			
Post-landmark follow-up duration, weeks	78 [48–164]	72 [22–153]	0.278
Subsequent relapse	4 (24)	18 (75)	–

Values are presented as mean ± standard deviation, median [interquartile range], or *n* (%), as appropriate. Continuous variables were compared using the Mann–Whitney U test, and categorical variables were compared using Fisher's exact test. Available-case denominators were used for variables with missing data. GC doses are expressed as prednisolone equivalents. Subsequent relapse was not subjected to between-group hypothesis testing because it was the outcome of the landmark survival analysis. *Among patients treated with tacrolimus. GC, glucocorticoid; ARS, aminoacyl-tRNA synthetase; ILD, interstitial lung disease; IS, immunosuppressive; TAC, tacrolimus; CRP, C-reactive protein; CK, creatine kinase; KL-6, Krebs von den Lungen-6.

Among the 22 patients with post-landmark first relapse, ILD was the most common manifestation (16 patients), followed by myositis (5 patients) and skin disease (1 patient). All four relapses in the GC ≤5 mg/day group involved ILD. Among the 18 relapses in the GC >5 mg/day group, 12 involved ILD, five involved myositis, and one involved skin disease.

### Cox proportional hazards analysis

All 41 patients and 22 post-landmark first-relapse events were included in the Cox proportional hazards analyses. In univariable analysis, GC ≤5 mg/day at week 26 was associated with a lower risk of subsequent relapse (HR 0.26, 95% CI 0.06–0.76; *p* = 0.014). Anti-Jo-1 positivity was also associated with a lower risk, whereas Raynaud phenomenon was associated with an increased risk (Table 3).

**Table 3:** Cox proportional hazards analysis of factors associated with relapse after week 26.

Variable	Univariable HR (95% CI)	p	Model 1 HR (95% CI)	p	Model 2 HR (95% CI)	p
GC ≤5 mg/day at week 26	0.26 (0.06–0.76)	0.014	0.27 (0.08–0.86)	0.027	0.32 (0.10–0.99)	0.047
Raynaud phenomenon	5.55 (1.85–16.65)	0.002	2.93 (0.91–9.46)	0.073	2.66 (0.84–8.44)	0.097
Anti-Jo-1 positive	0.27 (0.10–0.73)	0.009	—	—	0.35 (0.13–0.96)	0.042
Myositis	0.99 (0.42–2.33)	0.979	—	—	—	—
Initial GC dose, per 1 mg/day	0.99 (0.96–1.02)	0.391	0.98 (0.95–1.01)	0.268	—	—

Sensitivity analyses				
Model	GC ≤5 mg/day at week 26, HR (95% CI)	p	Covariate, HR (95% CI)	p
Adjusted for initial GC dose	0.21 (0.07–0.64)	0.006	Initial GC dose: 0.98 (0.95–1.01)	0.218
Adjusted for anti-Jo-1	0.27 (0.09–0.79)	0.017	Anti-Jo-1: 0.29 (0.11–0.77)	0.013

Additional sensitivity analysis	HR (95% CI)	p
GC dose at week 26, per 1 mg/day increase	1.04 (0.90–1.19)	0.611

Values are presented as HRs with 95% CIs. Cox proportional hazards analyses were performed in the week-26 landmark cohort (*n* = 41), with relapse after week 26 as the outcome. Model 1 included GC ≤5 mg/day at week 26, Raynaud phenomenon, and initial GC dose as a continuous variable. Model 2 included GC ≤5 mg/day at week 26, Raynaud phenomenon, and anti-Jo-1 positivity. GC doses are expressed as prednisolone equivalents. HR, hazard ratio; CI, confidence interval; GC, glucocorticoid; mPSL, methylprednisolone.

In Model 1, the association between GC ≤5 mg/day at week 26 and relapse persisted after adjustment for Raynaud phenomenon and initial GC dose (HR 0.27, 95% CI 0.08–0.86; *p* = 0.027). A similar association was observed in Model 2, which included Raynaud phenomenon and anti-Jo-1 positivity (HR 0.32, 95% CI 0.10–0.99; *p* = 0.047). Because of the small number of patients with Raynaud phenomenon, sensitivity analyses excluding this variable were performed. The association remained significant after separate adjustment for initial GC dose (HR 0.21, 95% CI 0.07–0.64; *p* = 0.006) and anti-Jo-1 positivity (HR 0.27, 95% CI 0.09–0.79; *p* = 0.017).

To examine the association without dichotomizing GC dose at 5 mg/day, week-26 GC dose was also analyzed as a continuous variable; no significant association with subsequent relapse was observed (HR 1.04 per 1 mg/day increase, 95% CI 0.90–1.19; *p* = 0.611).

## Discussion

In this retrospective cohort of anti-ARS antibody-positive patients with ASSD treated with GCs and concomitant immunosuppressive therapy, a GC dose ≤5 mg/day at week 26 was associated with a lower risk of subsequent relapse. To our knowledge, few studies have examined the association between GC dose during the early treatment course and relapse in ASSD under concomitant immunosuppressive therapy. The association remained statistically significant in both multivariable models, with similar effect estimates, and sensitivity analyses separately adjusting for initial GC dose and anti-Jo-1 positivity yielded consistent results. Nevertheless, this association should not be interpreted as demonstrating an independent protective effect of a GC dose ≤5 mg/day at week 26.

Many patients with IIM require prolonged GC treatment, and minimizing GC exposure while maintaining disease control remains an important therapeutic goal. GC-related adverse effects may emerge within the first several months of therapy (16), and bone loss has been reported to develop within the first 6 months after diagnosis in patients with IIM (15). Anti-ARS antibody-positive ILD generally responds to initial GC therapy but is characterized by frequent recurrence, supporting the use of concomitant immunosuppressive therapy (6, 17). In the present cohort, only 4 of 45 patients (8.9%) experienced relapse during the first 26 weeks, whereas 26 of 45 (58%) experienced relapse during overall follow-up. This contrast between relatively few early relapses and substantial long-term recurrence supports the clinical relevance of assessing disease control at approximately 6 months.

Previous studies in PM/DM-associated ILD have suggested that concomitant immunosuppressive therapy may reduce recurrence, particularly in anti-ARS antibody-positive disease (20). Observations in ANCA-associated vasculitis also suggest that lower GC exposure does not necessarily increase relapse risk (21, 22). Our findings are consistent with these observations and suggest that a lower GC dose during the early treatment course may be compatible with sustained disease control under concomitant immunosuppressive therapy. Nevertheless, the GC dose at week 26 was determined by the treating physician and may also reflect preceding disease activity and treatment response. Thus, the week-26 GC dose may serve as a marker of early disease control or treatment response.

In univariable analysis, Raynaud phenomenon and anti-Jo-1 positivity were associated with subsequent relapse, although both associations were attenuated after multivariable adjustment. Clinical phenotype and disease course in ASSD vary according to anti-ARS antibody specificity, with non-Jo-1 antibodies reported to be associated with distinct clinical phenotypes and, in some studies, more prominent or severe ILD (23–28). This heterogeneity may provide a clinical context for the lower relapse risk observed with anti-Jo-1 positivity in the univariable analysis. Raynaud phenomenon is also a recognized manifestation of ASSD and may reflect vascular or broader systemic autoimmune involvement (29, 30).

This study has several limitations. First, this was a retrospective, single-center study with a modest sample size and a limited number of post-landmark relapse events, restricting generalizability, statistical precision, and multivariable adjustment. Raynaud phenomenon was present in only five patients in the landmark cohort, all of whom subsequently relapsed, raising the possibility of sparse-data bias and model instability. Although sensitivity analyses excluding Raynaud phenomenon yielded similar results, the multivariable estimates should be interpreted cautiously.

Second, treatment and clinical assessment were not standardized. Treatment decisions were made at the discretion of the treating physicians; thus, patients receiving GC ≤5 mg/day at week 26 may have had better-controlled or more treatment-responsive disease. Post-landmark treatment also varied according to clinical course and was not modeled as a time-varying exposure, and tacrolimus exposure could not be quantitatively evaluated because trough measurements were not standardized. Therefore, residual confounding related to treatment before and after the landmark cannot be excluded. The predominant use of tacrolimus (43/45 patients) may also limit generalizability to other immunosuppressive regimens. Comprehensive and standardized assessment of disease activity was limited, including the lack of standardized pulmonary function testing and quantitative assessment of ILD on CT. Relapse was retrospectively assessed without prespecified standardized organ-specific criteria or independent adjudication, and treatment escalation formed part of the outcome definition. Although relapse assessment incorporated relevant clinical, laboratory, and imaging findings and consideration of alternative explanations such as infection, outcome ascertainment bias cannot be excluded.

Third, the landmark analysis was restricted to patients who remained relapse-free and under observation through week 26 and evaluated GC dose at a single time point. Week 26 was a clinically relevant but partly pragmatic landmark, and neither week 26 nor the 5 mg/day cutoff should be considered a biologically established threshold.

## Conclusion

Among patients with anti-ARS antibody-positive ASSD receiving concomitant immunosuppressive therapy who remained relapse-free and under observation through week 26, a GC dose ≤5 mg/day at week 26 was associated with a lower risk of subsequent relapse. Although these findings should not be generalized to all patients or interpreted as establishing an optimal tapering target, the GC dose at this landmark may serve as a clinically useful marker of early disease control or treatment response. Prospective studies are warranted to validate its clinical significance.

## Data Availability

The raw data supporting the conclusions of this article will be made available by the authors, without undue reservation.
